# Generation of Schubert polynomial series via nanometre-scale photoisomerization in photochromic single crystal and double-probe optical near-field measurements

**DOI:** 10.1038/s41598-020-59603-1

**Published:** 2020-02-17

**Authors:** Kazuharu Uchiyama, Hirotsugu Suzui, Ryo Nakagomi, Hayato Saigo, Kingo Uchida, Makoto Naruse, Hirokazu Hori

**Affiliations:** 1 University of Yamanashi, 4-3-11 Takeda, Kofu, Yamanashi 400-8511 Japan; 2 Nagahama Institute of Bio-Science and Technology, 1266 Tamura, Nagahama, Shiga 526-0829 Japan; 3 Ryukoku University, 1-5 Yokotani, Oe-cho, Seta, Otsu, Shiga 520-2194 Japan; 4 Department of Information Physics and Computing, Graduate School of Information Science and Technology, The University of Tokyo, 7-3-1 Bunkyo-ku, Tokyo, 113-8656 Japan

**Keywords:** Electrical and electronic engineering, Applied mathematics, Optoelectronic devices and components, Nanophotonics and plasmonics

## Abstract

Generation of irregular time series based on physical processes is indispensable in computing and artificial intelligence. In this report, we propose and demonstrate the generation of Schubert polynomials, which are the foundation of versatile permutations in mathematics, via optical near-field processes introduced in a photochromic crystal of diarylethene combined with a simple photon detection protocol. Optical near-field excitation on the surface of a photochromic single crystal yields a chain of local photoisomerization, forming a complex pattern on the opposite side of the crystal. The incoming photon travels through the nanostructured photochromic crystal, and the exit position of the photon exhibits a versatile pattern. We emulated trains of photons based on the optical pattern experimentally observed through double-probe optical near-field microscopy, where the detection position was determined based on a simple protocol, leading to Schubert matrices corresponding to Schubert polynomials. The versatility and correlations of the generated Schubert matrices could be reconfigured in either a soft or hard manner by adjusting the photon detection sensitivity. This is the first study of Schubert polynomial generation via physical processes or nanophotonics, paving the way for future nano-scale intelligence devices and systems.

## Introduction

Irregular time series play critical roles in information and communication technology today, including in secure information transfer^1,2^, Monte Carlo simulations^3^, and machine learning^4,5^. Physical processes in nature are interesting resources for providing irregular time series, including deterministic dynamics such as chaos^6^, rather than only truly random sequences such as those caused by single photons^7^. Indeed, chaotic lasers enable interesting functionalities ranging from ultrafast random number generation^8^ and photonic reservoir computing^9^ to decision-making, reinforcement learning^10^, and artificial data generation^5^. In the case of chaotic time series, a minute initial difference results in significantly different series, while the series share common attributes specified by the dynamics therein. It has been found that the high complexity and high correlation of deterministic chaos contribute significantly to value alignment and decision-making^10^.

In this study, we demonstrated the physical generation of irregular time series from near-field optical systems using photoisomerization in a photochromic crystal, which was observed using a double-probe scanning near-field optical microscope (SNOM). Specifically, we generated Schubert matrices corresponding to Schubert polynomials^11,12^ via photon transmission through a photochromic crystal photoisomerized on the nanometre scale combined with a simple photon detection protocol. A sequence of transmitted photons following the observed transmission probability pattern provides a photon source that is highly correlated with the photoisomerized nanostructure formed from a local excitation position. The spatial positions of the transmitted photons were related to the cell of Schubert matrices. To our knowledge, this is the first report of the utilization of Schubert polynomials for physical random sequence generation.

**Figure 1:**
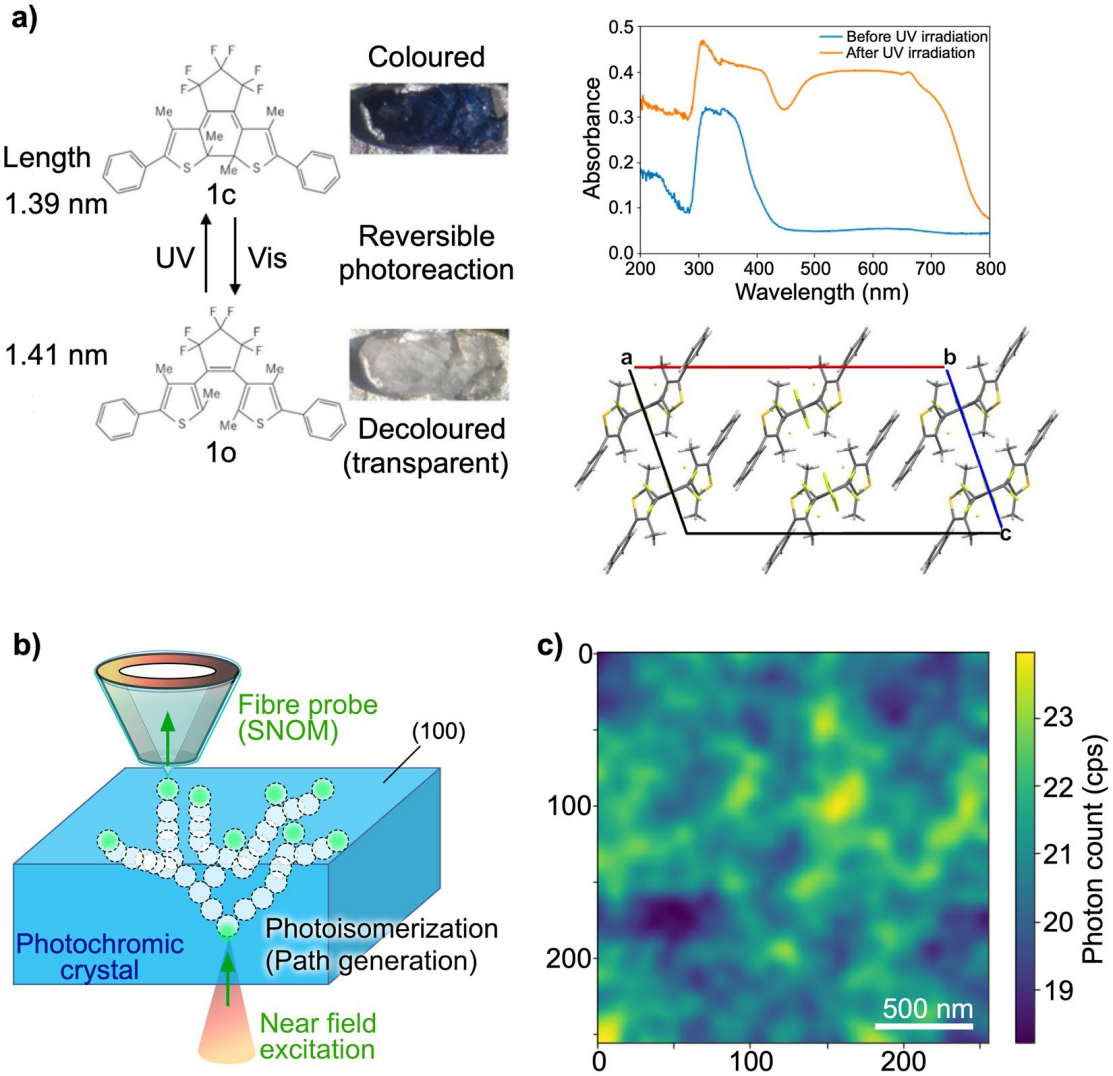
Double-probe SNOM measurement of photons propagating through the transparent paths in a photochromic crystal. (**a)** Diarylethene molecule structure and photoisomerization used in the present study. The absorbance spectra and crystal structure of the photochromic crystal are shown on the right side. **(b)** Experimental setup and schematic image of photon detection by double probe. **(c)** Map of optical near-field intensity.

The objective of this study was to produce highly correlated and yet significantly versatile time sequences, like those occurring in deterministic chaos dynamics. We employed optical near-field processes on the nanometre scale, where the physical system should obey certain constraints with the inherent nonlinearity. Our previous studies^13,14^ revealed that local optical near-field excitation on the surface of a photochromic crystal yields local photoisomerization on the scale of tens of nanometres. In this case, photochromic reaction between the colourless (transparent) open-ring isomer (**1o**) and blue-coloured closed-ring isomer (**1c**) proceeds in the crystalline state, and the coloured crystal upon ultraviolet (UV) irradiation was used as the recording material. The coloured material was decoloured due to cycloreversion reaction from **1c** to **1o** by the optical near field (Fig. 1a). Because the local transparency guides the optical near field into the deeper layer, the local photoisomerization to the transparent state induces a succeeding chain of local transparency including bifurcations, leading to the formation of a complex pattern on the *opposite* surface of the crystal, as shown in Fig. 1b. Figure 1c depicts one of the experimentally observed patterns measured by double-probe optical near-field microscopy^13^, where near-field excitation was performed at a specific single point on one surface of the photochromic crystal by a probe, while the other probe was scanned on the opposite side to observe near-field optical transmission through the entire thickness of the crystal.

## Results and Discussion

Here, we provide a brief review of the experiment conducted in our previous study^13^ to clarify the nature of the near-field optical pattern shown in Fig. 1c. The sample used in the experiment was a photochromic single crystal of a diarylethene (**1o**) with the molecular and crystal structures shown in Fig. 1a ^15–17^. Upon UV irradiation, the sample was converted into the blue coloured isomer (**1c**) with the crystal structure maintained^18–20^, exhibiting visible light absorption with a broad peak wavelength in the green to orange region. Upon visible light irradiation, the sample was converted into colourless isomers transparent to visible light. The spectra on the right side of Fig. 1a show the absorption spectra of the sample before and after UV irradiation. Both spectra have peaks near 350 nm in the UV region. The sample coloured using UV irradiation exhibits visible light absorption with a broad peak around 500–650 nm. The quantum yield of decolourization (cycloreversion, **1c** to **1o** (0.027)) is about 35 times less than that of colourization (cyclization, **1o** to **1c** (0.96))^17^. The single crystal used in this study was slab-shaped with the largest plane (the (100) surface) being a square with side lengths of approximately 0.5 mm and a thickness of about 0.1 mm (see the *Methods* section for details about the sample).

The measurements were conducted using a double-probe SNOM (UNISOKU, USM-1300S) on the (100) surface (see *Supplementary Information* for details about the crystal structure). The local optical excitation of the diarylethene surface was performed with a metallic probe tip, and optical near-field transmission measurement was conducted with an optical fibre probe (see the *Methods* section for details about the probes). The two probe tips were aligned so that their horizontal positions were separated by less than 2 μm prior to measurement^13^. The sample coloured via irradiation by UV light with a wavelength of 375 nm and an intensity of 5 mW for 30 min was inserted between the two position-adjusted probes, and the approach of the two probes toward the sample was controlled via scanning tunnelling microscopy (STM). The photoisomerization patterns generated by the local near-field light source at the metal probe tip (the wavelength of the light was 532 nm) were measured in a two-dimensional area with dimensions of 2 μm × 2 μm with a resolution of 256 pixels × 256 pixels (see the *Methods* section for details about the double probe measurement system).

Figure 1c shows the optical near-field intensity distribution obtained after adopting a two-dimensional Gaussian filter with a standard deviation of 6 pixels, which corresponds to the resolution limit of the system. A complex structural pattern is observable, with a representative scale of 100–200 nm. Specifically, the local near-field excitation (by the metallic tip) did not propagate uniformly through the photochromic crystal, but a chain of local photoisomerization with a physical scale less than 1/5 of the optical wavelength was induced, involving spontaneous symmetry breaking, as mentioned in the *Introduction* section.

**Figure 2:**
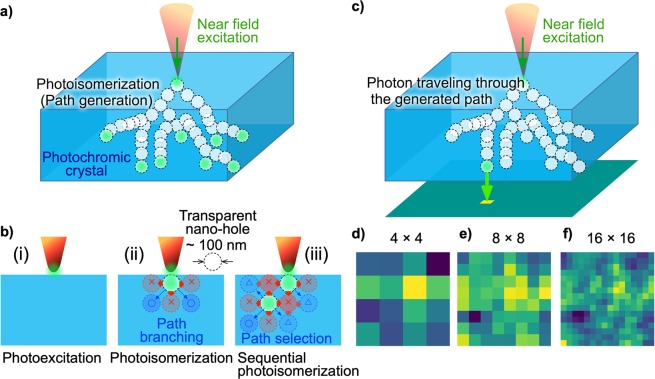
Photon source for generation of Schubert matrices with nanometre-scale photoisomerization in photochromic crystal and double-probe SPM measurements. (**a)** Formation of transparent path by photoisomerization with spontaneous symmetry breaking. **(b)** Chain of anisotropic local photoisomerizations from local photoexcitation. **(c)** Detection of photons propagating along the transparent path in a photochromic crystal for Schubert matrix generation. **(d–f)** Photon detection probability density maps with dimensions of 4 × 4, 8 × 8, and 16 × 16 generated by coarse graining of optical near-field images.

Such complex pattern generation has been considered to be due to a balance between the mechanical deformation of photochromic materials and photoisomerization^13,14^; that is, local photoisomerization induces anisotropic deformation of molecular size, leading to mechanical anisotropic strain^21,22^, which induces subsequent photoisomerization in a non-uniform manner in the surrounding material (Fig. 2a). Hence, spontaneous symmetry breaking is evident in the local photoisomerization involving branching and selection, as schematically depicted in Fig. 2b(i–iii). Firstly, as shown in Fig. 2b(i), optical near-field local excitation was applied by the probe tip to the surface of a photochromic crystal that was initially uniformly coloured by prior UV irradiation. The near-field-excited spot was locally made transparent. The generated transparent spot yielded a mechanical anisotropic strain in the adjacent region indicated by the ×-marks in Fig. 2b(ii), and the photoisomerization in the region was suppressed. The regions in which the suppression effect was weak, indicated by O-marks in Fig. 2b(ii), became the next candidates for photoisomerization, and branches for path formation were created (indicated by the two arrows in Fig. 2b(ii)). In other words, an anisotropic strain field from adjacent locally photoisomerized regions resulted in an anisotropic spatial distribution of the quantum yield of photoisomerization within the crystal. As illustrated in Fig. 2b(iii), when one of the branches was further selected, the additional anisotropic strain field overlapped so that the photoisomerization of the other branch was suppressed (as indicated by the ∆-marks) and other branches were generated. Such chains of photoisomerization and strain-field formation were stored in the photochromic crystal and provided nanometre-scale transparent paths, leading to versatile pattern generation, as observed in Fig. 1c. The typical size of the transparent path generated with branching and selection was estimated to be about 100 nm from the SNOM image^13^. The complex paths were formed over a sample thickness of 0.1 mm starting from the excitation point and were estimated to extend the same distance in the horizontal direction within the crystal. As mentioned above, the horizontal deviation between the two probe tips facing each other was less than 2 μm, which is sufficiently smaller than the spread of the paths, and the ends of the paths could be observed.

After transparent path generation, we used the photons traveling through the paths via optical near-field interaction from the probe tip as photon sources for Schubert matrix generation. In this study, we considered an input light irradiated by a near-field tip in a single photon level, with a photon energy lower than that necessary to induce further photoisomerization. Since the molecule employed does not show thermal relaxation from the closed- to open-ring isomer or vice versa^16,23^, the formed pattern is preserved until re-colouring with UV irradiation. The photon travels through the generated complex transparent paths and is transmitted from a certain exit position on the opposite side of the input near-field probe, as schematically depicted in Fig. 2c. The spatial position of the output differs photon by photon. By regarding each spatial position as a different code, a sequence of irregular codes can be generated.

Although the spatial position of the output photon is versatile, it is *not* arbitrary, because the transparent paths are produced in a specific manner in the path formation phase. Specifically, the geometry of the photon path in the photochromic crystal defines the underlying structure of the resulting code sequence.

In this study, instead of conducting single photon measurements with an array of photodetectors, we experimentally observed the photon statistics at each of the spatial positions by using the SNOM. The experimental data provide probability distributions that emulate single photon arrival. Depending on the size of the single photon source needed for application, we rescaled the optical near-field intensity image. Figure 2d–f show renormalized images with dimensions of 4 × 4, 8 × 8, and 16 × 16, respectively. Each pixel is a square with side lengths of 125 nm in the case of 16 × 16 resolution.

### Generation of Schubert polynomial series

Using this emulated photon source and a photon detection protocol, we created what we call *Schubert matrices*. A Schubert matrix is a matrix that represents the one-to-one associations between the row numbers (1 to *N*) and column numbers (1 to *N*) in an *N* × *N* matrix. When a cell (*i*, *j*) is 1, row *i* and column *j* are associated, and the other cells in the same row and column are 0. The total number of different Schubert matrices is *N*!. By replacing rows (or columns) of the matrix, the entire pattern can be generated. In other words, a Schubert matrix is a permutation matrix with respect to a diagonal matrix. A Schubert matrix has a one-to-one correspondence with a Schubert polynomial through divided difference operators^11,12^ (see the *Methods* section for details about the definition of Schubert polynomials).

**Figure 3:**
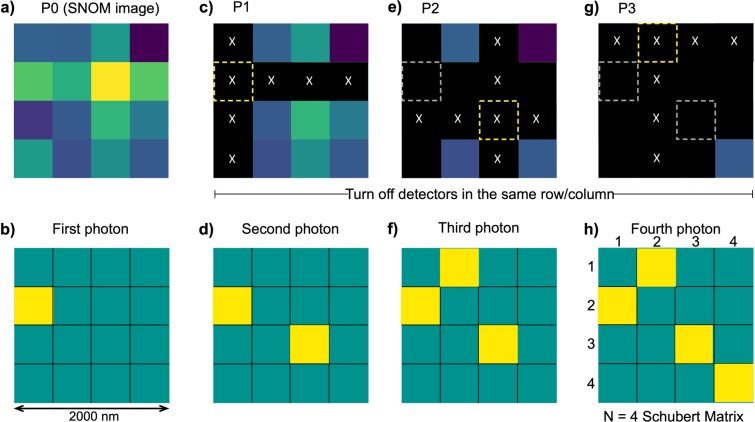
Method of Schubert matrix generation using an optical near-field intensity image as a probability density map of photon detection. (**a)** Optical near-field intensity image with dimensions of 4 × 4 used as a probability map. **(b)** First photon detected, whose position was stochastically determined using the probability map (represented by the yellow pixel). **(c**) Setting of the probability of points in the same row/column as the first photon to zero (black). **(d–g)** Process of sequential photon detection and probability changes. **(h)** Schubert matrix generated through processes **(a**–**g)**.

Figure 3 schematically shows how to generate a 4 × 4 Schubert matrix. Experimentally, we examined a square area with side lengths of 2 μm. The size of each pixel is about 500 nm in this configuration. The photon arrival probability is represented as an intensity distribution in Fig. 3a (see the *Methods* section for details regarding the conversion from intensity into probability). By using uniformly distributed pseudorandom numbers, the position of the first photon arrival was determined (Fig. 3b). Note that the maximum intensity position is not necessarily determined by the probabilistic nature of a single photon. According to the definition of a Schubert matrix, the probability of photon detection was configured to be zero for pixels whose row or column was the same as that of the first photon arrival location (Fig. 3c). Based on the reconfigured probability distribution, the position of the second photon arrival was determined (Fig. 3d). Again, the photon detection probabilities of the pixels located in the same row or column as the second photon were set to zero (Fig. 3e), followed by the determination of the third photon arrival position (Fig. 3f). The position of the fourth photon was determined automatically (Fig. 3g), because no two rows or columns could have photons. The resulting Schubert matrix is depicted in Fig. 3h. This pattern is represented by [2134], showing the column numbers of the photon detection pixels (coloured in yellow) in the order of the rows. The corresponding Schubert polynomial is *x*_1_ (see the *Methods* section for details regarding Schubert polynomials). There are 4! = 24 types of Schubert polynomials in total in the case of a 4 × 4 Schubert matrix, which is not a large number. However, the total number of possible polynomials grows exponentially as the size of the Schubert matrix increases. For a 16 × 16 Schubert matrix, there are 16! types of polynomials, which is on the order of 10^13^. Thus, extremely diverse sequences can be generated physically by optical near-field processes.

**Figure 4:**
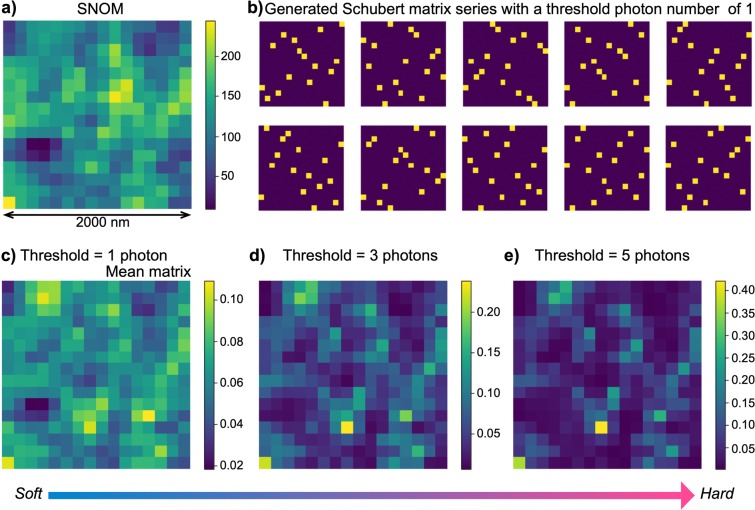
Generation of Schubert matrix series with different threshold photon numbers. (**a)** Observed SNOM image (same as in Fig. 2f). (**b)** First 10 generated Schubert matrices with a threshold photon number of 1. **(c–e)** Mean matrix of the 10,000 Schubert matrices generated with different threshold photon numbers: 1 photon for **(c)**, 3 photons for **(d)**, and 5 photons for **(e)**.

We obtained a series of Schubert matrices with dimensions of 16 × 16 using the abovementioned protocol. The side length of each cell was about 125 nm. According to our previous research^13,14^, the size of elemental photoisomerization was less than 100 nm; hence, a single pixel corresponded to at most a few terminals of the light paths. Figure 4 summarizes the results in the case of 16 × 16 resolution. Based on the SNOM image shown in Fig. 4a, 10,000 Schubert matrices were generated. Figure 4b depicts the first 10 matrices, where versatile patterns are observable. Figure 4c represents the mean of these 10,000 matrices. It should be noted that the mean matrix and original SNOM image have structural similarity but that the intensity distributions are very different because of the exclusive properties, meaning that only a single pixel occupies a given row and column in the process of Schubert matrix generation. Therefore, for example, if an SNOM image contains multiple high intensity pixels in a common row or column, the resulting Schubert matrix exhibits a diverse pattern.

As discussed above, the arrival of a single photon immediately provides an element in the Schubert matrix. By implementing an additional constraint on the photon detection, a Schubert matrix that is strictly correlated with the source SNOM pattern can be generated. We set a threshold for photon detection; that is, if the number of photons exceeded a certain threshold, an element of the Schubert matrix was determined. In this case, the high intensity positions in the SNOM images were highly likely to yield elements in the Schubert matrix. That is, the properties of the photochromic nanostructure were more strongly transferred to the resulting Schubert matrix.

Figure 4d,e present the mean Schubert matrices obtained from the original 10,000 matrices when the threshold level was set to 3 and 5 photons, respectively. Whereas the maximum value in the mean Schubert matrix is about 0.1 when the threshold is 1, the corresponding value increases to 0.2 and 0.4 in the cases of 3 and 5 photons, respectively. That is, a specific element is selected more often in the Schubert matrix as the threshold photon number increases. Therefore, the correlation between the optical near-field distributions and the resulting sequences can be tuned by the degree of photon detection; highly sensitive detection provides higher randomness or *softness*, while lower sensitivity yields limited versatility or *hardness*. It should be noted that the same optical near-field processes and the same measurement apparatus provide tunable or reconfigurable functionality, which would be useful for applications such as large-scale decision-making^24^.

**Figure 5:**
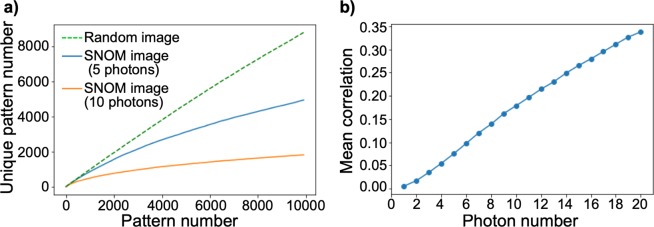
Properties of the generated Schubert matrices. (**a)** Time evolution of the number of unique Schubert matrices as a function of repetition cycle. The results obtained with thresholds of 5 and 10 photons are represented by blue and red lines, respectively. The corresponding results for randomly generated matrices are also presented, as a dotted line. **(b)** Mean correlations between pairs of all 10,000 Schubert matrices for different thresholds, from 1 to 20 photons.

We examined the diversity of the Schubert matrices, or their corresponding time sequences, when the SNOM image was rescaled to 8 × 8 because the maximum number of patterns is reasonable in that case (8! = 40,320). The number of unique Schubert matrices decreases as the photon number threshold increases. Figure 5a shows the number of unique Schubert matrices as a function of repetition cycle where, after 10,000 repetitions, the number of unique patterns is only 4925 and 1809 when the threshold is 5 and 10 photons, respectively. In the case of the Schubert matrices created by pseudorandom numbers, there were 8768 unique patterns out of 10,000 matrices.

Figure 5b summarizes the mean correlations between pairs among all 10,000 Schubert matrices. The correlation is nearly zero when the photon threshold is 1, while the correlation increases as the photon threshold increases; the correlation is more than 0.15 and 0.3 when the threshold is 10 and 20 photons, respectively. That is, tunable correlation was successfully accomplished by controlling the sensitivity of photon detection. It should be emphasized that, although the resulting patterns are extremely diverse, especially in the case of lower photon sensitivity, they are highly regulated by the SNOM images. In other words, they are not purely random, as is clearly evident from the mean structure of the resulting Schubert matrix in Fig. 4c.

Since local photoisomerization (decolourization) is performed while maintaining the symmetry of the original crystal^22–24^, it is possible to repeat the path formation by erasing (i.e. resetting) the formed transparent paths with colouring by UV irradiation. Since the path formation involves stochastic selection from branches of photoisomerization directions generated by anisotropic strain fields, a different path structure is formed each time. Different paths give Schubert matrix series with different correlations.

In this study, we generated Schubert matrices by utilizing a photon number profile observed by SNOM, which was later combined with a simple photon detection protocol that involves deactivating certain pixels. Direct detection of photons from photochromic materials by using a two-dimensional sensor and macroscopic parallel observation will be interesting topics of future study from a technological standpoint. The photon detection protocol could be implemented in hardware by utilizing intelligent vision chip technologies^25^.

The time required to generate a Schubert matrix by measurement using a photon detection array can be reduced in proportion to the number of detections. The current photon detection rate is expected to be about 300 cps for 1 pixel in the case of *N* = 16, where the generation of a matrix by 16 photons using a 16 × 16 detection array takes about 0.05 s. Since such measurement with a detection array is free from probe-scanning architecture, it is considered possible to perform generation with short latency, which is anticipated to be particularly effective when the threshold value is set high.

Finally, we describe possible solutions for generating larger Schubert matrices. The local photoisomerization size in this study was about 100 nm, which is close to 125 nm, when the SNOM image was divided into 16 × 16 pixels. The first solution entails obtaining an SNOM image in a wider area. Another solution involves using a different molecular crystal in which the molecules show larger deformations in photoisomerization^14^. To implement the second solution, measurement resolution improvement is also necessary.

In conclusion, we proposed and demonstrated the generation of Schubert polynomials via optical near-field processes introduced in a photochromic crystal of diarylethene combined with a simple photon detection protocol. The local photoisomerization induced in the photochromic crystal provided a complex, non-uniform internal structure, leading to a variety of photon transmission pathways. We employed two-dimensional photon detection protocol and successfully generated diverse patterns by introducing non-sensitive portions into the measurements concerning the definition of Schubert polynomials. We also demonstrated that, by changing the photon detection sensitivity, the variety of the resulting sequences as well as their correlations with one another can be configured. In other words, the relations between the generated Schubert polynomials and nanostructure properties can be tuned, which is important for future applications in which autonomous adaptation to the environment is crucial, such as in decision-making and soft robotics^24,26^.

The light transmission through the complex paths in a nanomaterial involves a quantum structure; a photon travels the superposition of multiple paths but converges to a certain single observation. Thus, this system could be an interesting and important platform for quantum measurement^27^. Furthermore, *multiple* local input excitations of the material, unlike the *single* local excitation approach employed in the present study, could provide higher functionality, such as enhanced versatility. As discussed in the *Introduction* section, the demand for irregular time series for use in information security and artificial intelligence, among other fields, is increasing. The fusion of advanced mathematics and physical processes including nanophotonics could be an interesting and exciting interdisciplinary topic for future research.

## Methods

### Sample preparation

The molecule used in this study was a diarylethene, 1,2-bis(2,4-dimethyl-5-phenyl-3-thienyl)perfluorocyclopentene. The single crystal employed was obtained by recrystallization from methanol. The crystal was colourless and thus consisted of open-ring isomers. The X-ray crystallographic data are as follows: monoclinic, space group *C2/c*, Z = 4, *a* = 24.023(4) Å, *b* = 8.4660(15) Å, *c* = 13.350(2) Å, *β* = 109.235(3)° ^21^. On the front and rear sides of the sample (the (100) surface), a thin Pt layer approximately 10 nm thick was coated to make the surface conductive, because the position of our optical near-field probe tip was controlled by utilizing an STM.

### Probe preparation

A metallic probe tip for local optical excitation was sharpened using an electropolishing method to a radius of curvature of several tens of nanometres and coated with an approximately 20-nm-thick Au layer. This metal probe was illuminated by 532 nm laser light with an intensity of 1 μW/cm^2^ to generate an optical near field by local electric field enhancement at the tip. The optical fibre probe was obtained by sharpening the optical fibre using a buffered hydrofluoric acid solution followed by coating with 10-nm-thick Pt.

### Double probe measurement system

The double-probe measurement system used in this study was based on an ultra-high vacuum high magnetic field STM-controlled SNOM system (UNISOKU USM-1300S)^13^. The measurements were taken at the bottom part of the SNOM head insert set into the superconducting magnet, and the view ports of the vacuum chamber were shielded during the experiment so that the sample was shielded from external light. Photons captured by the optical-fibre probe were counted with a photomultiplier tube (Hamamatsu Photonics H7422P-40). The SNOM measurements took about 7 h.

### Schubert polynomial

The four-dimensional Schubert matrix (that is, the four-dimensional symmetric group ${S}_{4}$) and corresponding Schubert polynomial (${{\mathfrak{S}}}_{\omega },\omega \in {S}_{4}$) are explained below as an example^11,12^. A four-dimensional identity matrix can be expressed as [1234] (*id*). The anti-identity matrix [4321] (${\omega }_{0}$) is the permutation of the longest length (6) in ${S}_{4}$ (‘length’ is the number of simple transposition cycles necessary to generate the permutation from *id*). The Schubert polynomial for ${\omega }_{0}$ is ${{\mathfrak{S}}}_{{\omega }_{0}}\equiv {x}_{1}^{3}{x}_{2}^{2}{x}_{3}$ (in the *n*-dimensional Schubert matrix, ${{\mathfrak{S}}}_{{\omega }_{0}}\equiv {x}_{1}^{n-1}{x}_{2}^{n-2}\cdots {x}_{n-2}^{2}{x}_{n-1}$). Other polynomials corresponding to matrices $\omega$ are determined by divided difference operators corresponding to necessary simple transpositions to convert to the anti-diagonal matrix (${\omega }^{-1}{\omega }_{0}$). One matrix [4312] becomes [4321] by exchanging rows 3 and 4 (this simple transposition is represented as ${s}_{3}$). Thus, the Schubert polynomial corresponding to [4312] is ${\partial }_{{\omega }^{-1}{\omega }_{0}}{{\mathfrak{S}}}_{{\omega }_{0}}\,=\,{\partial }_{3}{{\mathfrak{S}}}_{{\omega }_{0}}\,=\,({{\mathfrak{S}}}_{{\omega }_{0}}-{s}_{3}{{\mathfrak{S}}}_{{\omega }_{0}})/({x}_{3}-{x}_{4})\,=\,({x}_{1}^{3}{x}_{2}^{2}{x}_{3}-{x}_{1}^{3}{x}_{2}^{2}{x}_{4})/({x}_{3}-{x}_{4})={x}_{1}^{3}{x}_{2}^{2}$. Other examples include ${{\mathfrak{S}}}_{id}\,=\,1$, ${{\mathfrak{S}}}_{1432}\,=\,{\partial }_{1}{\partial }_{2}{\partial }_{3}{{\mathfrak{S}}}_{{\omega }_{0}}\,=\,{x}_{1}^{2}{x}_{2}+{x}_{1}^{2}{x}_{3}+{x}_{1}{x}_{2}^{2}+{x}_{1}{x}_{2}{x}_{3}+{x}_{2}^{2}{x}_{3}$, and ${{\mathfrak{S}}}_{2431}\,=\,{\partial }_{1}{\partial }_{2}{{\mathfrak{S}}}_{{\omega }_{0}}\,=\,{x}_{1}^{2}{x}_{2}{x}_{3}\,+\,{x}_{1}{x}_{2}^{2}{x}_{3}$.

### Conversion of optical near-field intensity map into probability map

We let the intensity distribution of the obtained near-field light be *NF* (*x*, *y*) (*x* = 1,…, *N*, *y* = 1,…, *N*, where *N* is the pixel size of the SNOM image). The value obtained by subtracting 0.99 times the minimum intensity was defined as *NFS* (*x*, *y*) = *NF* (*x*, *y*) − min (*NF* (*x*, *y*)) × 0.99. Since the near-field light intensity contained background noise, 0.99 times the minimum value was subtracted as noise in this case. The probability distribution was given by *P* (*x*, *y*) = *NFS* (*x*, *y*)/Σ *NFS* (*x*, *y*). In addition, for pixels whose sensitivity was made 0 in the process of deriving the Schubert matrix, the update *NFS* (*x*, *y*) = 0 was made and *P* (*x*, *y*) = *NFS* (*x*, *y*)/Σ *NFS* (*x*, *y*) was recalculated (see the *Supplementary Information* for details regarding the conversion).

## Supplementary information

Supplementary information.

## Data Availability

The datasets generated during the current study are available from the corresponding author on reasonable request.
