# Supplementary material for: Generation of Schubert polynomial series via nanometre-scale photoisomerization in photochromic single crystal and double-probe optical near-field measurements
Source: Sci Rep. 2020 Feb 17;10:2710. doi: 10.1038/s41598-020-59603-1 (PMC7026093; doi:10.1038/s41598-020-59603-1)
Supplement: Supplementary file 1 — Supplementary information. [file 41598_2020_59603_MOESM1_ESM.pdf]

Supplementary Information

Article in *Scientific Reports*

Generation of Schubert polynomial series via nanometre-scale photoisomerization in photochromic single crystal and double-probe optical near-field measurements

Kazuharu Uchiyama, Hirotsugu Suzui, Ryo Nakagomi, Hayato Saigo, Kingo Uchida, Makoto Naruse, and Hirokazu Hori

In this supplementary information, we present the experimental details.

1. Absorption spectra of the used photochromic molecule

The absorption spectrum of the molecule **1c** (closed-ring isomer) as hexane solution reported in [1] (Fig. 1) had a peak near 560 nm. The absorption spectrum of the coloured single crystal reported in [2] (Fig. 2b) had a peak near 630 nm. Both of them had absorption peaks at 532 nm and were used in this study as excitation light.

2. Crystal structure of the employed sample

The sample used in this study is the crystal of open-ring isomer show in Fig. 1a. A three-dimensional schematic diagram of the molecular packing is provided in Fig. S1.

3. Conversion of optical near-field intensity map into probability map.

In this study, when converting a near-field intensity image into a probability map, we subtracted 0.99 times the minimum intensity from the near-field intensity image as background noise. In the previous study ([14]), we performed SNOM measurements without optical-near-field excitation (Fig. S2a) and optical-near-field excitation (Fig. S2b), and the measurements results are provided in Figs. S2c and S2d (the same image as Fig. 1c), respectively. The histograms of the photon counts of these images are shown in Fig. S2e. The background noise in the SNOM image taken with the optical near-field could be estimated between V_A: lowest intensity of the image taken with far-field excitation (~17.0 cps) and V_B: the minimum value of the image used for Schubert matrix generation (18.6 cps in the case of $N = 16$). When we subtracted $V_B \times 1.00$, there were pixels that were never chosen as elements of the Schubert matrices. We subtracted $V_B \times 0.99$ (~18.4 cps) so that every point could be a matrix element. Figure S3a shows the mean matrix of 10,000 Schubert matrices in the case of 18.4 cps subtraction, and Figure S3b shows that in the case of 17.0 cps subtraction. The patterns are similar and the probability contrast is slightly higher when 18.4 cps is subtracted. From the above, although the determination of the value to be subtracted from the SNOM image has a certain range, if it is selected within this range, the main point of this paper is not affected.

References

1. Irie, M., Sakemura, K., Okinaka, M. & Uchida, K. Photochromism of diarylethenes with electron-donating substituents. *J. Org. Chem.* **60**, 8305 (1995).
2. Shibata, K., Muto, K., Obatake, S. & Irie, M. Photocyclization/cycloreversion quantum yields of diarylethenes in single crystals. *J. Phys. Chem. A* **106**, 209 (2002)

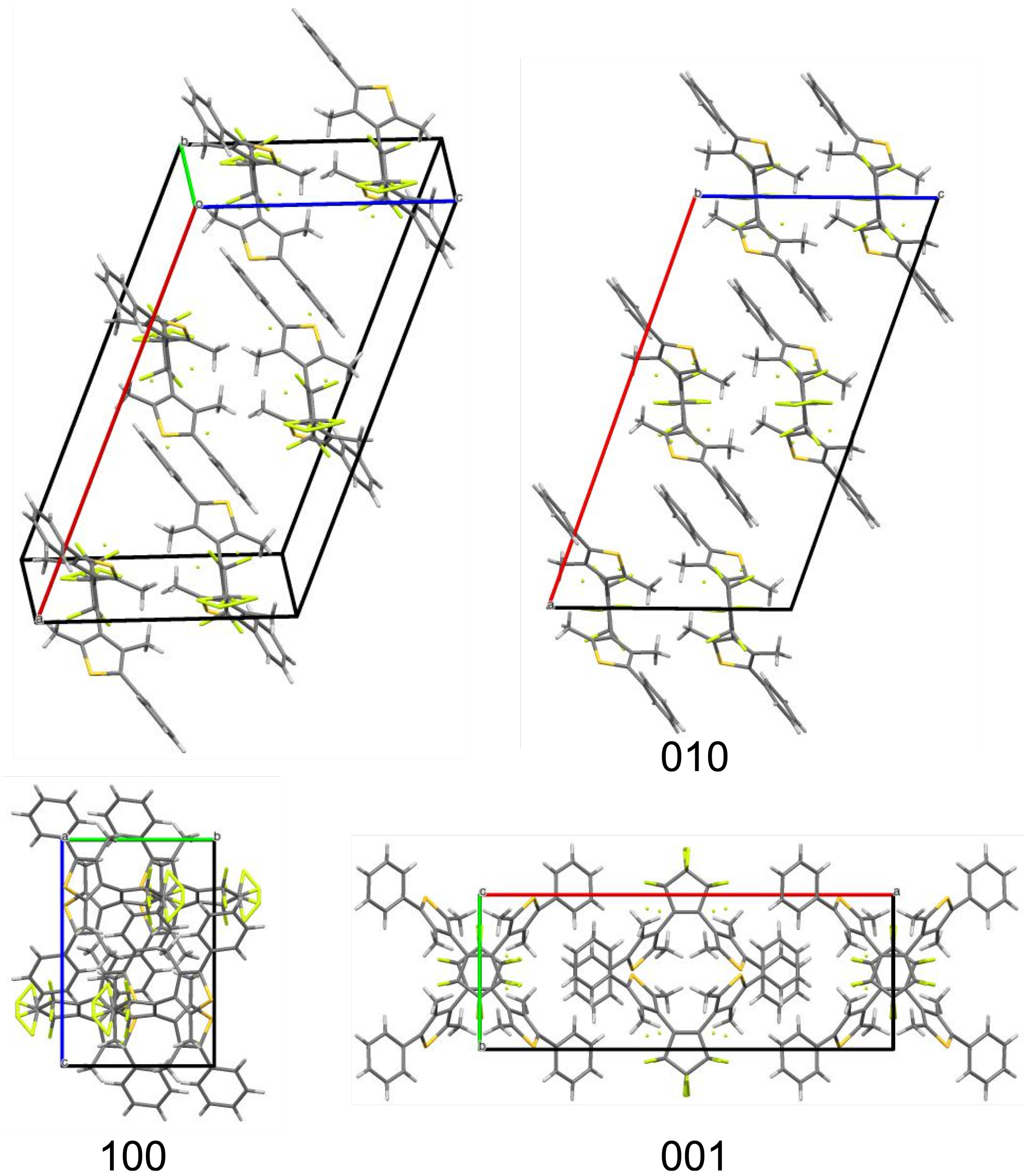

Fig. S1 Molecular packing the crystal of open-ring isomer **1o** prepared by recrystallization.

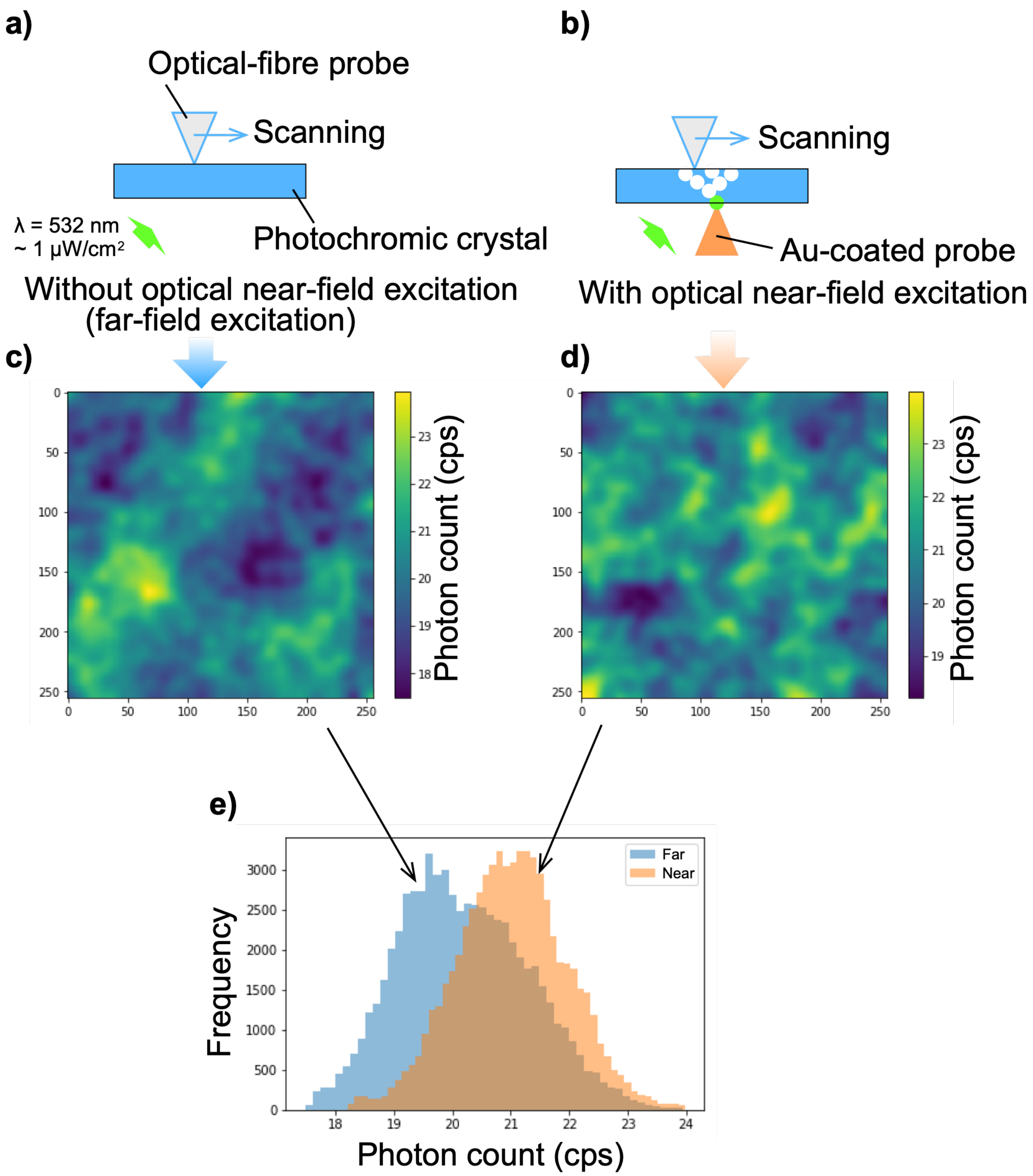

Fig. S2 (**a**) and (**b**) Experimental setups for SNOM measurement without and with optical near-field excitation. Molecular packing the crystal of open-ring isomer **1o** prepared by recrystallization. (**c**) and (**d**) SNOM images corresponding to (**a**) and (**b**), respectively. (**e**) Histograms of the intensities in (c) and (d).

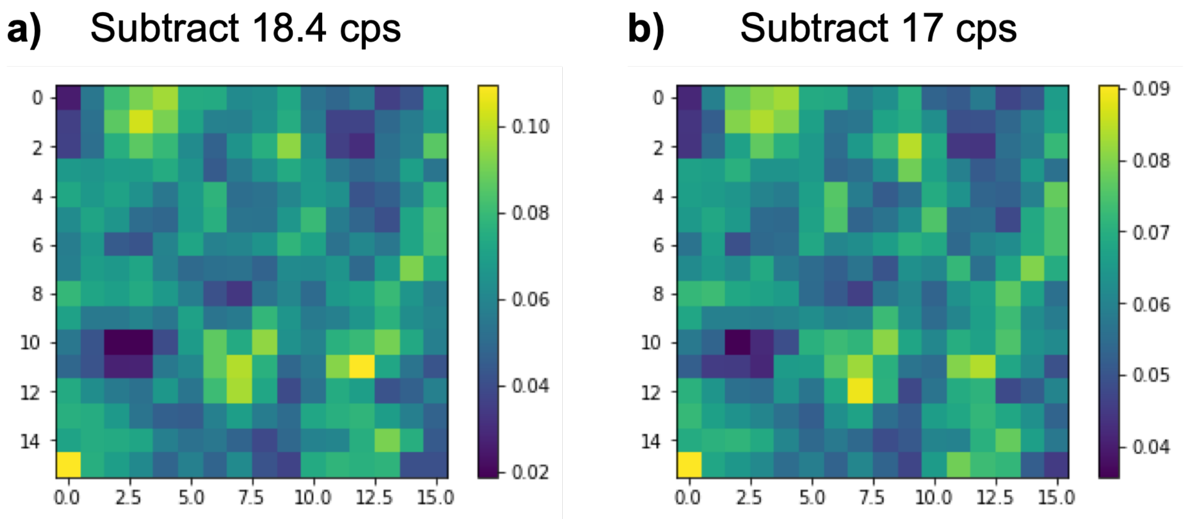

Fig. S3 Mean matrices of 10,000 Schubert matrices obtained with the intensity image subtracted by (**a**) 18.4 cps and (**b**) 17 cps.
